# Reducing *Baylisascaris procyonis* Roundworm Larvae in Raccoon Latrines

**DOI:** 10.3201/eid1701.100876

**Published:** 2011-01

**Authors:** Kristen Page, James C. Beasley, Zachary H. Olson, Timothy J. Smyser, Mark Downey, Kenneth F. Kellner, Sarah E. McCord, Timothy S. Egan, Olin E. Rhodes

**Affiliations:** Author affiliations: Wheaton College, Wheaton, Illinois, USA (K. Page, M. Downey, S.E. McCord);; Purdue University, West Lafayette, Indiana, USA (J.C. Beasley, Z.H. Olson, T.J. Smyser, K.F. Kellner, T.S. Egan, II, O.E. Rhodes, Jr)

**Keywords:** Baylisascaris, prevention, zoonoses, raccoon, roundworms, parasites, Indiana, dispatch

## Abstract

*Baylisascaris procyonis* roundworms, a parasite of raccoons, can infect humans, sometimes fatally. Parasite eggs can remain viable in raccoon latrines for years. To develop a management technique for parasite eggs, we tested anthelmintic baiting. The prevalence of eggs decreased at latrines, and larval infections decreased among intermediate hosts, indicating that baiting is effective.

The emergence of zoonotic diseases, which account for ≈58% of all infectious diseases in humans, is linked to changing land use and resource consumption patterns (1). Ecosystem disturbances from human population growth and globalization result in rapid spread of zoonotic pathogens (2). Recent integrated approaches to solving global health issues acknowledge that wildlife reservoirs facilitate zoonotic pathogen emergence and emphasize the need for increased collaboration between the ecology and infectious disease communities (2). We describe a multidisciplinary collaboration that used an experimental approach to lower the prevalence, and possibly break the life cycle, of a zoonotic parasite, the *Baylisascaris procyonis* roundworm.

## The Study

Raccoons (*Procyon lotor*) are the host of *B. procyonis* roundworms, intestinal parasites (3). Up to 82% of adult raccoons and 90% of juvenile raccoons are infected (3). Mature worms produce thousands of eggs daily (3). These eggs are eliminated through raccoon feces and accumulate at raccoon latrines (4). *B. procyonis* roundworm eggs remain infective for many years and can infect juvenile raccoons and intermediate hosts such as rodents and birds that ingest them (3). Transmission often occurs at raccoon latrines when eggs are ingested with seeds found in fecal material (4). Larvae migrate through intermediate host tissues and can enter the central nervous system, resulting in death (3). Adult raccoons become infected when they prey on infected intermediate hosts (3). Raccoon population densities have increased in response to increased anthropogenic resources that are available in agricultural and urban ecosystems (5). Thus, raccoon latrines often exist near human habitats, increasing the risk for zoonoses (4).

Reported cases of human *B. procyonis* roundworm infections are rare (n = 18), and all have occurred in North America; however, prevention is a public health priority because of the severity of the resulting neurologic disease (6–10). Our objective was to develop a management technique that could interrupt transmission of *B. procyonis* roundworm eggs between raccoons and intermediate hosts, ultimately decreasing the environmental levels of eggs and potential for reinfection. We examined the effects of latrine removal and treatment of raccoons by using randomly distributed anthelmintic baits on the basis of *B. procyonis* roundworm prevalence at latrines and among intermediate hosts. By implementing a specific, protocol-based approach to disease prevention, supported by experimentally derived data, we hope to provide public health officials with an effective, spatially explicit, prophylactic method for reducing infection risk.

We conducted this study in Grant, Miami, and Wabash counties in north-central Indiana in portions of the Upper Wabash Basin. This area is 88% agricultural; only 8% of the landscape remains forested (11). Some contiguous riparian forest remains; however, most patches are <5 hectares (ha; 1 ha = 10,000 m^2^) (11). Our experiment was conducted in 16 forest patches (1.91–8.80 ha). Eight treatment patches received anthelmintic baits, and 8 control patches did not. The range of patch sizes, levels of patch isolation, and raccoon densities in treatment and control patches were representative of the landscape (Figure).

**Figure:**
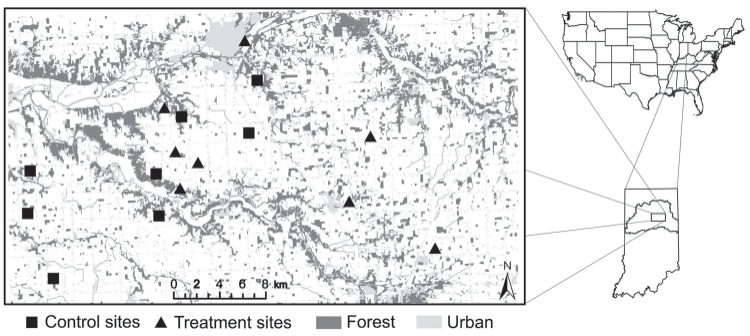
Study area of raccoon latrines showing locations of treatment and control patches, Upper Wabash Basin, north-central Indiana, 2007–2008. Dominant land use is represented by degree of shading.

In March 2007 (spring 07), we removed all visible latrines (n = 559) in the treatment patches. We located latrines by systematically searching all appropriate horizontal substrate and area at the bases of large trees throughout each forest patch (*3*). After manual removal, we used a torch to sterilize the substrate and surrounding soil associated with each latrine (Technical Appendix). At control sites, we sampled a minimum of 20 latrines (n = 198) by removing ≈2 g fecal material per fecal deposit at each latrine (12). We returned to our study sites 3 additional times for fecal sampling in October and November 2007 (fall 07), June 2008 (summer 08), and November 2008 (fall 08). During these subsequent visits, we sampled ≈2 g of fecal material per fecal deposit at a minimum of 20 latrines in all treatment and control patches. All samples were stored at −20°C until they were examined for *B. procyonis* roundworm eggs*.* Eggs were identified by microscopic examination following centrifugal fecal flotation in Sheather sugar solution (3). We identified *B. procyonis* roundworm eggs on the basis of size and morphologic appearance and designated each sample as positive or negative. Prevalence was measured as the proportion of positive samples at each study patch during each sampling period. Differences between pretreatment and posttreatment prevalence and between treatment and control patches were determined by using log linear analyses performed with PROC CATMOD SAS version 9.1 (SAS Institute Inc., Cary, NC, USA) (goodness-of-fit tests).

In spring 07, after the initial latrine removal from treatment patches, baits were distributed throughout treatment patches once a month for the duration of the study. Baiting densities were determined on the basis of average abundance of raccoons in each study patch (Technical Appendix).

Prevalence of *B. procyonis* roundworm larvae within an intermediate host, white-footed mice (*Peromyscus leucopus*), was determined. A minimum of 10 mice were captured from each of the 16 study patches during each of 3 sampling periods: 1 pretreatment (summer 07), and 2 posttreatment (fall 07 and summer 08). After capture, mice were euthanized with carbon dioxide and refrigerated until examination for *B. procyonis* roundworm larvae. Brains were removed and examined separately by pressing them between glass plates, and larvae were examined under a dissecting microscope. We recovered larvae from tissues digested in acid–pepsin solution (3). Larvae were counted and identified (3). Prevalence of infection was determined for mice within each study patch for each sampling period. Differences between treatment and control patches were determined by Fisher exact test (12).

We collected 1,797 fecal samples. Pretreatment sampling of latrines in spring 07 detected *B. procyonis* roundworm eggs at 757 (33%) of latrines sampled across all patches (Table). However, prevalence of eggs in treatment patches declined by >3-fold after baiting in all sampling periods (p<0.04). Our baseline pretreatment estimate of prevalence of infection among intermediate hosts did not differ (p = 0.426) between treatment patches (32%) and control patches (37%). Approximately 1 year after baiting activities began, we detected a significant decline in the prevalence of *B. procyonis* roundworm larvae in mice between treatment and control patches (27% vs. 38%; p = 0.05; Table).

**Table:** Findings from baited patches in study of prevalence of *Baylisascaris procyonis* roundworms at raccoon latrines and among intermediate hosts, Upper Wabash Basin, north-central Indiana, 2007–2008*

Patch	1	2	3	4	5	6	7	8
Control patches								
Size, ha	8.19	6.67	2.95	1.91	6.67	6.43	4.28	4.39
Raccoons/ha	1.22	1.35	1.69	3.66	2.25	1.56	1.17	3.42
Baits/ha	0	0	0	0	0	0	0	0

Prevalence at latrines								
Spring 07	0.36	0.17	0.43	0.06	0.45	0.27	0	0.38
Fall 07	0.65	0.05	0	0.13	0.13	0.24	0	0.15
Summer 08	0.19	0	0.08	0.05	0	0.05	0	0
Fall 08	0.40	0.13	0.03	0.10	0.40	0.50	0.07	0.47
Total change	0.11	−0.24	−0.93	0.67	−0.11	0.85	NC	0.24

Prevalence among intermediate hosts								
Summer 07	0.25	0.42	0.35	0.38	0.09	0.27	0.56	0.54
Fall 07	0.27	0.28	0.50	0.22	0.18	0.38	0.45	0.25
Summer 08	0.44	0.33	0.53	0.55	0.56	0.25	0.05	0.37
Total change	0.76	−0.21	0.51	0.44	5.22	−0.07	−0.91	−0.32

Treatment patches								
Size, ha	9.66	6.72	4.72	2.50	2.46	3.66	6.00	8.80
Raccoons/ha	1.40	1.19	1.91	2.00	4.47	3.83	4.00	1.70
Baits/ha	7.00	5.95	9.53	10.00	22.36	19.12	20.00	8.52
Prevalence at latrines								

Spring 07	0.12	0.64	0.08	0.38	0.38	0.29	0.05	0.52
Fall 07	0	0.05	0	0.18	0.15	0.17	0.18	0
Summer 08	0	0	0	0	0.05	0	0	0
Fall 08	0.17	0	0.03	0.03	0.30	0.17	0	0.03
Total change	0.42	−1.00	−0.63	−0.92	−0.21	−0.41	−1.00	−0.94
Prevalence among intermediate hosts								
Summer 07	0.28	0.50	0.25	0.30	0.25	0.33	0.18	0.33
Fall 07	0.47	0.47	0.58	0.50	0.36	0.45	0.25	0.40

Summer 08	0.25	0.10	0	0.35	0.40	0.45	0.22	0.33
Total change	−0.11	−0.80	−1.00	0.17	0.60	0.36	0.22	0

*Prevalence, proportion of positive samples (parasites present). The effect of treatment on prevalence by patch is represented by the proportional change between pretreatment and final sampling. ha, hectare (10,000 m^2^); spring 07, March 2007; fall 07, October and November 2007; summer 08, June 2008; fall 08, November 2008; NC, no change.

## Conclusions

Current public health initiatives to prevent human infections with *B. procyonis* roundworms focus on education of human health care and veterinary professionals (6). Our practical approach decreased prevalence of the parasite, suggesting decreased transmission and possibly reduced risk for humans. Baiting strategies have effectively controlled rabies (13) and decreased prevalence of zoonotic parasites, including *Echinococcus multilocularis* tapeworms (14). Our baiting strategy combined with latrine removal effectively decreased egg levels at latrines and ultimately decreased prevalence among mice. Hegglin and Deplazes (14) demonstrated a long-term decrease in prevalence of *E. multilocularis* tapeworms among foxes (definitive hosts) after monthly baiting for ≈4 years and conjectured that this decrease was caused by decreased infections among intermediate hosts. Our study supports their hypothesis because we measured decreases in prevalence among intermediate hosts after baiting. The reduction of prevalence at latrines and among intermediate hosts suggests that our low-cost approach (Technical Appendix) could have a lasting effect on transmission dynamics; however, further study to assess frequency of distribution and type and dose of baits for sustained prevalence is needed. Raccoon latrines are commonly found near homes (4), and implementation of baiting strategies, in conjunction with traditional raccoon management on public lands, could reduce the risk for transmission on nearby private properties.

## Supplementary Material

Appendix Latrine Removal and Baiting protocols.
