# Supplementary material for: Reducing Baylisascaris procyonis Roundworm Larvae in Raccoon Latrines
Source: Emerg Infect Dis. 2011 Jan;17(1):90–3. doi: 10.3201/eid1701.100876 (PMC3204634; doi:10.3201/eid1701.100876)
Supplement: Appendix — Latrine Removal and Baiting protocols. [file 10-0876-Techapp.pdf]

Article DOI: 10.3201/eid1701.100876

Reducing *Baylisascaris procyonis* Roundworm Larvae in Raccoon Latrines

Technical Appendix

Latrine Removal Protocol

We manually removed all visible latrines (n = 559) in our 8 treatment sites. Following removal, we used a Red Dragon Vapor Torch (BP 2512 SVC; Red Dragon Back Pack Kit with squeeze valve/handle kit; Flame Engineering, LaCrosse, Kansas, USA) to sterilize the substrate and surrounding soil associated with each latrine. The flame was kept on the substrate until soil or substrate was red to white-hot. This process required a second person to carry a backpack water tank that was used to extinguish any flames, thus reducing risk of fire.

Baiting Protocol

Baiting commenced in treatment patches immediately following latrine removal. Baiting densities were determined based on average abundance of raccoons in a study patch during the period of 2004–2006. Patch-specific raccoon density estimates were calculated based on the average number of raccoons captured within each patch during ongoing mark-recapture experiments divided by the area of the forest patch (www.berrymaninstitute.org/journal/fall2008/Beasley_Rhodes.pdf). We distributed baits at a rate of 5 baits/estimated raccoon, thus bait densities in our study patches ranged from 25 (5 raccoons/patch) to 120 baits (24 raccoons/patch; Table). Baits consisted of a fishmeal polymer attractant identical to those employed in the Oral Rabies Vaccination program (Bait-tek, Orange, TX, USA) and contained pyrantel pamoate (Strongid Paste, Pfizer, New York, NY, USA) at a dose of 3mg/0.454kg of estimated average body weight (average raccoon body weight in our study site = 4.5kg), suspended in a mixture of 1.83 g of marshmallow cre`me (Kroger, Cincinnati, OH, USA) and 0.135 mL of nanopure water to facilitate bait acceptance. We sealed the pyrantel pamoate suspension within the fishmeal attractant with paraffin wax. Individual baits cost ≈$0.50, and can be assembled at a rate of 100/hour.
